# Delaying pigs from the normal production flow is associated with health problems and poorer performance

**DOI:** 10.1186/s40813-017-0061-6

**Published:** 2017-07-05

**Authors:** Julia Adriana Calderón Díaz, Alessia Diana, Laura Ann Boyle, Finola C. Leonard, Máire McElroy, Shane McGettrick, John Moriarty, Edgar García Manzanilla

**Affiliations:** 1 Pig Development Department, Teagasc Grassland Research and Innovation Centre, Moorepark, Fermoy, Co. Cork, Ireland; 2 Department of Animal Behaviour and Welfare, Institute of Genetics and Animal Breeding, Polish Academy of Sciences, Jastrzębiec, Magdalenka, Poland; 3 School of Veterinary Medicine, University College Dublin, Dublin, Ireland; 4 Central Veterinary Research Laboratory, Department of Agriculture, Food and the Marine Laboratories, Backweston, Celbridge, Co. Kildare, Ireland

**Keywords:** All-in/all-out, Performance, Pigs, Production flow, Respiratory diseases

## Abstract

**Background:**

Delaying pigs from advancing through the production stages could have a negative impact on their health and performance. The objective of this study was to investigate the possible implications of delaying pigs from the normal production flow on pig health and performance in a farrow-to-finish commercial farm with a self-declared All-In/All-Out (AIAO) management.

**Results:**

Three flows of pigs were defined, flow 1 (i.e. pigs that followed the normal production flow; 8 weeks in the nursery stage, 4 weeks in the growing stage and 8 weeks in the finisher stage), flow 2 (i.e. pigs delayed 1 week from advancing to the next production stage) and flow 3 (i.e. pigs delayed >1 week from advancing to the next production stage). Flow 3 included higher proportions of pigs from first parity sows and of lighter birth weights. When the 3 flows were matched by parity and birth weight, pigs in flow 2 were 3.8 times more likely to be lame prior to slaughter compared with pigs in flow 1. Similarly, pigs in flow 3 were more likely to be lame prior to slaughter, 4.5 times more likely to present pleurisy, 3.3 times more like to present pericarditis and 4.3 times more likely to have their heart condemned at slaughter compared with pigs in flow 1. Additionally, carcasses from pigs in flow 3 were 10 kg lighter compared with carcasses from pigs in flow 1.

**Conclusion:**

Delayed pigs were more affected by disease and were lighter at slaughter. Besides animal welfare issues, these findings could represent considerable economic loses for pig producers. In practice, delaying pigs from the normal production flow translates into higher feeding costs, increase number of days to slaughter and increased labour requirements reducing production efficiency for the pig operation. In farrow-to-finish farms an ‘all-forward’ policy (i.e. no pig is left behind from stage to stage and a split marketing approach is applied when sending pigs to slaughter) might be more easily adhered to.

## Background

All-In/All-Out (AIAO) production systems are widely used in the pig industry as they reduce disease transmission and improve management and growth performance [1, 2]. In a true AIAO system, groups of pigs are moved together to the next production stage and the facility is completely emptied, cleaned and disinfected before the next group arrives. As groups are closely matched by age, body weight (BW) and production stage, uniformity in terms of growth and feed efficiency is expected. Nevertheless, natural variation in growth rates of pigs means that the slower-growing animals may pose management challenges in AIAO systems. For ease of management and contrary to a true AIAO system, slower growing or smaller animals may be held back to a similarly sized following batch of younger animals. This risks disease transmission from older to younger animals, as such between batches, and thus increases likelihood of disease spread and occurrence [3]. Thus, the objective of this study was to investigate the possible association between delaying pigs from the normal production flow and pig health and performance in a farrow-to-finish pig farm with self-declared AIAO management.

## Methods

The study was conducted on a 1500 sow farrow-to-finish commercial farm in Ireland, positive for enzootic pneumonia and swine influenza and negative for PRRS. Details regarding animal management and measurements recorded are described in Calderón Díaz et al. (*submitted*). In brief, a total of 1016 pigs born within one week were individually tagged at birth and followed to slaughter. Sex, number of piglets born alive, sow parity, number of times each piglet was cross-fostered and lactation length were recorded. This farm purported that it followed an AIAO policy whereby pigs spend 8 weeks in the nursery stage after weaning, 4 weeks in the growing stage and 8 weeks in the finisher stage. Animals were managed as per usual practice on the farm (for more details please refer to Calderón Díaz et al., *submitted*) and the weekly movement of animals was tracked. 18.9% of pigs died during the study. One- hundred-and-﻿ four pigs died during the lactation period, 24 pigs died during nursery, 3 pigs died during growing and 14 pigs died during the finishing stages. Forty-seven pigs were selected for euthanasia on the basis of showing external lesions and/or pathologies such as hernias, severe tail biting (i.e. complete tail loss), severe lameness, external abscesses, emaciation etc. for a study investigating respiratory pathologies. Details on reasons for euthanasia and results for the study regarding respiratory pathologies will be presented in a separate manuscript.

Eight-hundred-and-twenty-four pigs reached slaughter age. All animals were slaughtered within 1 week, regardless of their body weight, at 24 weeks of age and were retrospectively classified into three production flows according to the time they spent in each production stage [i.e. flow 1 = normal (*n* = 620 pigs), flow 2 = delayed by 1 week (*n* = 111 pigs) and flow 3 = delayed by >1 week (*n* = 93)].

Prior to slaughter, pigs were scored for lameness by a single trained observer (AD) on a 3-point scale where 1 = non lame; 2 = mildly lame and 3 = severely lame. At slaughter, tail lesions were scored after scalding and dehairing by one trained observer (as per [4]). Cold carcass weight, fat thickness and muscle depth were recorded by the slaughterhouse personnel. Percentage of lean meat was calculated according to the formula established by the European Communities Pig Carcass Grading Amendment Regulations [5].

$$ \%\mathrm{lean}\ \mathrm{meat}=60.30-\left(0.847\times \mathrm{fat}\ \mathrm{thickness}\right)+\left(0.147\times \mathrm{muscle}\right) $$

Pleurisy was scored using the Slaughterhouse Pleurisy Evaluation System (SPES; [6]) and Enzootic pneumonia (EP) like lesions were scored according to the BPEX Pig Health Scheme [7] by a trained observer (MME). Additionally, presence or absence of pericarditis and condemnations of the heart and liver were recorded.

### Data management and statistical analysis

Each pig was considered as the experimental unit. As only one pig was scored as severely lame, lameness was re-classified into non-lame and lame. Tail lesions, pleurisy and EP were also re-classified as present or absent. All data were analysed in SAS v9.3 (SAS Inst. Inc., Cary, NC). ANOVA tests for sow parity, birth weight and number of piglets born alive were conducted including data from all animals in the batch that reached slaughter (*n* = 824 pigs) to confirm that these parameters were not different between flows. Statistical differences were detected for parity and birth weight between flows and therefore, a nested case control design was employed whereby pigs from the three flows were matched by sow parity, birth weight and number of piglets born alive. The final data set included 120 pigs in flow 1, 60 pigs in flow 2 and 60 pigs in flow 3.

Univariable models, with flow as a predictor variable, were used to investigate the relationship between flow and the recorded variables. Alpha level for determination of significance and trends were 0.05 and 0.10, respectively. Lameness, tail lesions, pleurisy, EP, pericarditis and heart and lung condemnations were analysed in PROC GENMOD using binomial logistic regression. Cold carcass weight, muscle and fat content and lean meant percentage were analysed using linear model equations in PROC MIXED.

## Results

**Fig. 1:**
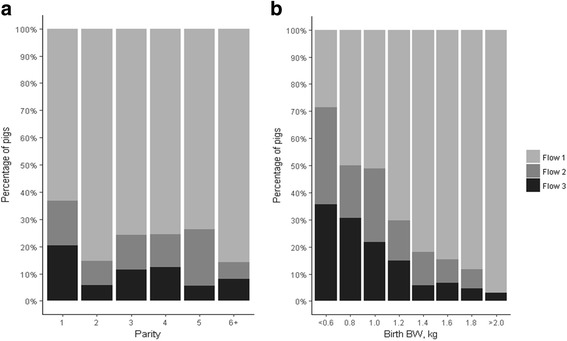
Percentage of pigs by (**a**) parity and (**b**) birth body weight in three different production flows. One batch of pigs born within one week were followed from birth to slaughter in a farrow-to-finish commercial farm. All animals were slaughtered within 1 week at 24 weeks of age and were retrospectively classified into three production flows (i.e. Flow 1 = normal, Flow 2 = delayed by 1 week and Flow 3 = delayed by >1 week) according to the extra time they required to be moved to the next production stage

For the analysis including all the pigs, mean parity was lower in flow 3 (2.9 ± 1.50) compared with mean parity in flow 1 (3.4 ± 1.43) and flow 2 (3.3 ± 1.49) (*P* < 0.05) with a greater percentage of pigs born from first parity sows in flow 3 (28.6%) than in flow 1 (13.4%) and flow 2 (19.4%; Fig. 1a). Similarly, mean BW at birth was lower for pigs in flow 3 (1.19 ± 0.30 kg) than for pigs in flow 1 (1.44 ± 0.28 kg) and flow 2 (1.26 ± 0.29 kg) (*P* = <0.001; Fig. 1b) with 5.6% of pigs in flow 1; 14.4% of pigs in flow 2 and 24.7% of pigs in flow 3 having a birth BW of <0.95 kg. In a separate analysis, the latter weight, < 0.95 kg, was identified as the threshold for a higher risk of mortality during the production cycle in this population (see Calderón Díaz et al., *submitted*). No differences were identified between flows for litter size (14.2 ± 2.9 piglets in flow 1; 14.6 ± 2.5 piglets in flow 2 and 14.0 ± 2.5 in flow 3; *P* > 0.05).

**Fig. 2:**
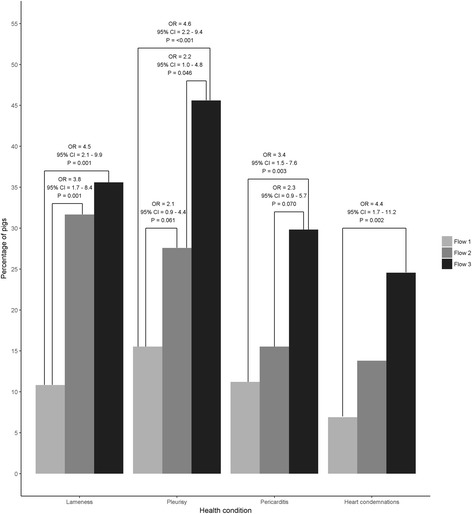
Percentage of pigs, odds ratios (OR) and 95% confidence interval (CI) for lameness, pleurisy, pericarditis and heart condemnations. The figure includes 240 finisher pigs from one batch born within one week that was followed from birth to slaughter in a farrow-to-finish commercial farm. All animals were slaughtered at 24 weeks of age and were retrospectively classified into three production flows (i.e. Flow 1 = normal, Flow 2 = delayed 1 week and Flow 3 = delayed >1 week) according to the time they required to be moved to the next production stage. Pigs were selected from each flow in a nested case control study matched by parity and birth weight

The odds ratios (OR) for the likelihood of lameness, pleurisy, pericarditis and heart condemnations for pigs matched by birth weight and parity in each flow are presented in Fig. 2. Pigs in flow 2 had greater odds of lameness and tended to have greater odds of pleurisy compared with pigs in flow 1 (*P* < 0.05). Pigs in flow 3 had greater odds of lameness, pleurisy, pericarditis and heart condemnations compared with pigs in flow 1 (*P* < 0.05). Additionally, pigs in flow 3 had greater odds of pleurisy and tended to have greater odds of pericarditis compared with pigs in flow 2 (*P* < 0.05). No differences between flows were found for the percentage of pigs with tail lesions (64.3% in flow 1; 63.9% in flow 2 and 55.4% in flow 3; *P* > 0.05) and EP (43.6% in flow 1; 47.2% in flow 2 and 48.3% in flow 3; *P* > 0.05). However, it is important to note that the majority of the tail lesions were mild.

The least square means for the carcass characteristic traits by flow are presented in Table 1. Pigs in flow 3 had lighter carcasses, less muscle depth and less fat thickness compared with pigs in flow 1 (*P* < 0.05). There was no difference in muscle depth and fat thickness between pigs in flow 2 and pigs in flow 3 (*P* > 0.05). Additionally, there was no difference in lean meat % between flows (*P* > 0.05).

**Table 1:** Univariable linear models for the association between three different production flows and cold carcass weight, lean meat %, muscle depth and fat thickness in 240 finisher pigs selected for a nested case control design

	Cold carcass weight		Lean meat %^2^		Muscle content, kg		Fat thickness, mm	
Production flow^1^	LS mean	SE	LS mean	SE	LS mean	SE	LS mean	SE
1	88.54^a^	0.90	56.65^a^	0.20	52.91^a^	0.38	13.48^a^	0.23
2	86.18^a^	1.27	56.56^a^	0.28	52.26^a,b^	0.54	13.49^a,b^	0.33
3	78.48^b^	1.27	57.18^a^	0.28	49.11^b^	0.54	12.21^b^	0.33

^1^All animals were slaughtered within 1 week at approximately 20 weeks post-weaning and were retrospectively classified into three production flows according to the extra time they required to be moved to the next production stage (i.e. Flow 1 = normal, Flow 2 = delayed 1 week and Flow 3 = delayed >1 week)

^2^Calculated according to the formula established by the European Communities Pig Carcass Grading Amendment Regulations (Department of Agriculture, Fisheries and Food, 2001) as %lean meat = 60.30 − (0.847 × fat thickness) + (0.147 × muscle)

^a,b^Within columns, significant differences between levels of each predictor variable; *P* < 0.05

## Discussion

An AIAO production system improves pig performance and health [1] but it poses challenges for the management of slow growing pigs. In fact, a disadvantage of an strict AIAO system is the growth variation which is associated with inefficient pen utilisation [8] and poor carcass grading [9]. In this study, 11% of pigs were in flow 3 which is a similar to the percentage of pigs previously reported as slow growing at market age in AIAO systems [10–12]. In an AIAO system, slow growing pigs should only be delayed from the normal production flow ‘off-site’ [2] but this does not always happen in practice and producers need to find alternative management practices that improve productivity without compromising biosecurity and vice versa. The farm where this study was conducted self-declared as following an AIAO system but in fact there was a continuous flow of animals through the system with smaller pigs being delayed from moving to the next production stage, in some cases, by several weeks. Although this may have been inadvertent (e.g. because the farmer believed that the slow growing pigs were younger pigs), it could also be that slow growing pigs are repeatedly delayed to allow them to catch up and reach adequate slaughter weights. This seems to be a quite common practice in Irish farms and probably in other countries. In fact, we have observed the same practice on a separate study where seven farms are being followed longitudinally, with all the farms delaying pigs from the normal production flow although they declare to follow an AIAO system. Pig producers must adhere to specific BW range specifications at the time of slaughter in order to avoid financial penalties imposed by the abattoir if pigs fall outside the range [13]. It has been reported that price per kg of dead weight can decrease up to 60% if pigs are too light [14]. Thus, by delaying pigs from the normal production flow and regrouping them based on BW, producers expect to reduce BW variation and economic loses. However, slow growing pigs require 4 to 6 weeks longer to reach adequate slaughter weights [8] increasing production cost and the possibility of poor carcass grading due to higher fat content [10, 15] and studies have shown that re-grouping slow growing pigs does not improve their growth performance [16, 17] mainly due to the fact that slow growing pigs would still receive the same feed and management practices. Nonetheless, segregating slow growing pigs removes competition from heavier pen mates and offers the opportunity to implement management practices such as greater feeder space and specialise diets that could help to improve their growth performance and reduce the extra time needed to reach adequate slaughter weights.

In this study we identified three clear flows of animals, those moved to the next stage as planned (flow 1), those delayed one week at some point in the production cycle (flow 2) and those delayed repeatedly (flow 3). Early life indicators (e.g. sow parity and birth BW) differed between these 3 production flows. Pigs in flow 3 were born from lower parity sows and had lower birth weights compared with pigs from flow 1 and flow 2. Such factors were associated with an increased likelihood of poor health and performance (see Calderón Díaz et al., *submitted*). Pigs born from younger sows are more susceptible to disease (Calderón Díaz et al., *submitted*) primary due to the lower availability of good quality colostrum and milk [12, 18] which translates into lower passive immunity [19]. Similarly, lighter pigs at birth also have poorer immune development [19] making them more prone to die during lactation [20], be more susceptible to infection agents [3] and more likely to show poorer growth rates [21]. Hence, these differences in early life indicators between pigs in the 3 flows were at least partly responsible for some of the related health issues. However, we were interested in determining whether there was an association between delaying pigs from advancing through the production stages per se and poorer health and performance over the production cycle. The nested case-control design allowed us to match pigs by parity and birth weight to investigate differences in pig health and performance associated with the production flow independently of the other underlying factors.

Our results confirm the association of production flow with the likelihood of disease. Pigs that did not follow the normal production flow had a higher level of disease, supporting the theory that delaying pigs from advancing through the production stages is associated with the re-circulation of disease and a higher risk of exposure to pathogens [22]. Pleurisy and pericarditis are some of the main reasons for carcass condemnations related to infection in slow growing pigs [23]. Additionally, delaying animals probably leads to the transmission of pathogens to younger apparently healthy animals in the group that older delayed pigs join [3]. The difference between the flows in lameness levels could also be related to infectious causes but may also be indicative of other issues like aggression by re-mixing [24].

Slowly growing pigs affected by disease are also less feed efficient and may never reach the target slaughter weight [2]. In this study, in spite of the extra time the pigs were allowed before advancing to the next production stage, carcasses from pigs in flow 3 were 10 kg lighter which has serious economic implications for pig producers. Assuming a production cost of €1.46 per kg of meat produced and a price of €1.52 per kg of meat paid by the meat processing plant (values based on Irish averages obtained from the Teagasc eProfit Monitor for 2015), this represents a loss in revenue of €6.7 per pig excluding costs associated with extra labour, medication and feed, and the cost of having a pig space occupied for a longer time by the same animal.

Nonetheless, further research is needed to elucidate whether the greater risk of disease in delayed pigs are causative or explanatory. There are a number of potential scenarios which need to be investigated. For example, pigs are often delayed because they are assumed to be slow-growing, but they are in fact simply smaller and are even potentially healthy. These may acquire certain diseases/conditions as a consequence of being held back from the normal production flow (reverse biosecurity). For others; disease as the likely cause of slower growth and delay may be a disease and production risk to the new batch to which they have entered. Conditions such as lameness are likely to be causative and explanatory. Lame animals may be delayed to allow them to recover but it is also likely that animals delayed for other reasons are remixed several times increasing their likelihood of becoming lame [24]. Therefore, controlled studies should be carried out where seemingly healthy animals are delayed to try to explain this complex relationship. If studies are to be conducted in commercial farms, the original reasons for delaying pigs should be recorded.

## Conclusion

Delaying pigs from the normal production flow was associated with negative consequences for pig health and performance and could represent considerable disease risk and production loss for pig producers, if it is a common practice on the farm. Implementing a strict AIAO policy in a farrow-to-finish unit is difficult due to the variable number of weaners produced every week, the management of pig spaces available to maximise production and the financial penalties impose by abattoirs for sending light weight pigs to slaughter. Thus, it is possible that in a farrow-to-finish farm an ‘all-forward’ policy might be more easily adhered to whereby no pig is left behind from stage to stage but rather they are split marketed at the point of slaughter and slow growing pigs are allow to remain the extra time required to reach adequate slaughter weight only during the finishing stage.
